# Stability of building gene regulatory networks with sparse autoregressive models

**DOI:** 10.1186/1471-2105-12-S13-S17

**Published:** 2011-11-30

**Authors:** Jagath C Rajapakse, Piyushkumar A Mundra

**Affiliations:** 1 BioInformatics Research Centre, School of Computer Engineering, Nanyang Technological University, Singapore 639798; 2 Singapore-MIT Alliance, Singapore 639798; 3 Department of Biological Science, Massachusetts Institute of Technology, Cambridge, MA 02142, USA

## Abstract

**Background:**

Biological networks are constantly subjected to random perturbations, and efficient feedback and compensatory mechanisms exist to maintain their stability. There is an increased interest in building gene regulatory networks (GRNs) from temporal gene expression data because of their numerous applications in life sciences. However, because of the limited number of time points at which gene expressions can be gathered in practice, computational techniques of building GRN often lead to inaccuracies and instabilities. This paper investigates the stability of sparse auto-regressive models of building GRN from gene expression data.

**Results:**

Criteria for evaluating the stability of estimating GRN structure are proposed. Thereby, stability of multivariate vector autoregressive (MVAR) methods - ridge, lasso, and elastic-net - of building GRN were studied by simulating temporal gene expression datasets on scale-free topologies as well as on real data gathered over Hela cell-cycle. Effects of the number of time points on the stability of constructing GRN are investigated. When the number of time points are relatively low compared to the size of network, both accuracy and stability are adversely affected. At least, the number of time points equal to the number of genes in the network are needed to achieve decent accuracy and stability of the networks. Our results on synthetic data indicate that the stability of lasso and elastic-net MVAR methods are comparable, and their accuracies are much higher than the ridge MVAR. As the size of the network grows, the number of time points required to achieve acceptable accuracy and stability are much less relative to the number of genes in the network. The effects of false negatives are easier to improve by increasing the number time points than those due to false positives. Application to HeLa cell-cycle gene expression dataset shows that biologically stable GRN can be obtained by introducing perturbations to the data.

**Conclusions:**

Accuracy and stability of building GRN are crucial for investigation of gene regulations. Sparse MVAR techniques such as lasso and elastic-net provide accurate and stable methods for building even GRN of small size. The effect of false negatives is corrected much easier with the increased number of time points than those due to false positives. With real data, we demonstrate how stable networks can be derived by introducing random perturbation to data.

## Background

Biological networks are constantly perturbed randomly and there exist efficient and compensatory mechanisms to withstand such instabilities. Constructing gene regulatory networks (GRN) from time-series gene expression data plays a vital role in understanding complex biological mechanisms and in the development of novel drugs. Though microarrays allow measurement of thousands of genes simultaneous, gene expressions in practice can be gathered only over a few time points due to high cost and time involved, and limitations of the experiments. This makes building GRN inherently an ill-posed problem in practice, leading such networks unstable and irreproducible. Moreover, variable and complex nature of biological sources, and measurement noise and artifacts, add to the challenges of constructing accurate and stable GRN.

A wide range of techniques for inferring GRN from microarray datasets have been proposed in the literature [1-4], including Boolean networks [5], linear networks [6,7] differential equation models [8], and stochastic methods [9-12]. Despite the plethora of such techniques, stability of building GRN from gene expression data has not been addressed by the research community so far. In this paper, we investigate the stability of building GRN by using sparse linear models with simulations and real data.

The linear multivariate vector autoregression (MVAR) provides a simple and efficient technique to estimate regulatory relationships among genes. However, due to less number of time points compared to the number of genes whose expressions involved in gene expression datasets, several penalized MVAR techniques using regularization [3,7,13-16] and priors [17] have been considered for building GRN. The sparse MVAR techniques such as lasso and elastic-net uses a penalty function to drive small regulatory coefficient to zero, thereby producing computationally stable and sparse yet biologically plausible GRNs. Recently, their efficacy in building GRN have been demonstrated [6,7].

A good technique of building GRN should not only be accurate but also be reproducible and stable; biologists will then be able to test complex hypothesis with confidence on *in silico* networks. Stability means that the network construction is robust to changes of network topology and parameters, and biological and instrumental noise. In this paper, we first introduce novel criteria for evaluating stability of building GRN at the level of connections and networks. MVAR models of ridge, lasso, and elastic-net penalty are evaluated with respect to their accuracies and stabilities by using synthetic gene expression datasets. In particular, we investigate how many time points of gene expressions are needed for a network of given topology and size. Using a real data set gathered in HeLa cell cycle [18], we demonstrate how random perturbations in the data could be induced to determine stable genes in the network.

## Methods

Suppose dataset *X* = {*x_i_*(*t*)}_*I* × *T*_ consists of time-series of expressions of *I* genes, taken over equally spaced *T* time points, where *x_i_*(*t*) denotes the expression of gene *i* at time *t*. Let vector  represent expressions of all genes at time *t*. Consider a network of *I* genes, represented by an *r*-order multivariate vector autoregressive (MVAR) model:(1)

where  denotes the matrix of regression coefficients corresponding to a model of order *τ*, and  denotes residuals that are assumed to be i.i.d. and zero mean Gaussian. The regression coefficient  represents the interaction between genes *i* and *j*. In general, the model has *I*^2^*r* coefficients. This scope of this work is restricted to first-order systems (i.e., *r* = 1). Suppose a vector of gene expressions at time *t* is denoted by row vector  and let *z^t^* = *y*^*t*–1^ denote the vector of gene expressions at the previous time-point, *β* = [*β*^1^, *β*^2^, … *β^I^*]^T^ a matrix of size *I* × *I* of regression coefficients, and *ε^t^* = [*ε*_1_(*t*), *ε*_2_(*t*),…*ε_I_*(*t*)] the corresponding innovations. The multivariate model can be written in standard multivariate vector autoregressive (MVAR) form:(2)

In vector form, (2) becomes:(3)

where *t*-th row of *Y*, *Z*, and *E*, are *y^t^*, *z^t^*, and *ε^t^*, respectively, and there are *T* – 1 samples; *Y* is a (*T* – 1) × *I* matrix, *Z* a (*T* – 1) × *I* matrix, *β* a *I* × *I* matrix, and *E* a (*T* – 1) × *I* matrix. MVAR coefficients are estimated using standard least squares as:(4)

The above matrix cannot be determined if the number of genes are larger than number of samples (that is, *T* <<*I*).

To handle such instances, penalized regression methods such as ridge regression, lasso, or elastic net regression penalties have been proposed [19]. The general penalized regression loss function is given by:(5)

where λ and *α* are regularization parameters. When *α* = 0 the loss function represents ridge MVAR, *α* = 1 represents lasso, and *α* ∈ (0,1) represents an elastic-net penalty. In other words, lasso uses *L*_1_-norm, ridge regression uses L_2_-norm, and elastic-net uses both *L*_1_-norm and *L*_2_-norm in its penalty term. The penalty terms attempt to drive small regression coefficients to zero, making networks sparse and computationally well-posed. The lasso penalty is more capable of driving regression coefficients to zero than the ridge penalty.

### Synthetic data

Most of the real world networks, such as biological networks, are scale-free. In this study, GRN of various sizes and topology are built with scale-free networks using Barabasi-Albert model [20]. First, assuming that large GRNs adhere to the topology of scale-free networks, synthetic structures of GRN were built by initiating a small number of nodes. New nodes and edges were added with preferential attachment as the probability of addition of new nodes to the existing node is not uniform. A node with high number of edges attracts higher number of new nodes compared to a node with no connection. This phenonmena in fact leads to power-law distribution: the probability *p_i_* of preferential attachment of a new gene to existing gene *i* is given by:(6)

where d_i_ denotes the number of adjacent edges not initiated by gene *i* itself (which approximates to the in-degree of gene *i*). The parameters γ denotes the power of preferential attachment and *b* the attractiveness of the gene with no adjacent edges.

Time-series datasets of gene expressions were generated for a specific network topology with the preferential attachment γ = 1.2. For a given network topology, the coefficients corresponding to no interactions among genes were set to zero. For other connections, true MVAR coefficients were obtained by drawing samples from a uniform distribution on the interval [-1,-0.8] and [0.8,1] such that the number of positive and negative coefficients are approximately equal. The initial (*t* = 0) gene expression values were drawn from a uniform distribution on the interval [10,15]. For successive time points, expression were generated using MVAR model with added i.i.d. Gaussian random noise Σ = **I**[7]. Parameters used for generating synthetic networks are given in Table 1. For a given network and number of time points, we generated 100 time-series datasets by randomly initializing the gene expressions.

**Table 1:** Parameters used for generation of synthetic network

Parameter	Values
Number of Bootstraps (*B*)	100
Number of Genes	10, 50, or 100
Number of time points	10, 30, 50, 70
Regression coefficient cut-off for edge detection (*∈*)	0.0001

### Real data

To investigate to the stability of real biological networks, we use lasso and elastic-net methods on Human cancer cell Line (HeLa) cell-cycle gene expression dataset [18]. The dataset used in this study is described as experiment 3 (http://genome-www.stanford.edu/Human-CellCycle/Hela/) and contains 48 time points where gene expressions were measured at the interval of one hour and synchronized by double thymidine block. Based on relevance to cell cycle and tumor development, 91 genes were selected. This dataset was previously used to demonstrate the efficacy of sparse lasso MVAR techniques in building GRN [6]. In order to investigate stability random perturbations, we added noise perturbations at each time point by adding random samples from Gaussian noise *N*(0, (*σ* + *δ*)^2^); we define  where *σ* is the standard deviation of residuals of real data and *δ* is a perturbation constant. For real dataset, the standard deviation of residuals was, *σ* = 0.225 and *σ* = 0.23, respectively, for models built with lasso and elastic-net MVAR models. By generating 100 gene expression datasets for each of *δ* such that SNR ∈ [0.01, 4], the stability of GRN built were studied against noise perturbations.

### Stability of structure

In this section, we present a Hamming distance based criteria for evaluating the stability of building GRN. Let  be a set of *B* sub-samples of original data and *s^b^* be the GRN derived from *b*-th sample *X^b^*. The structure of each network *s^b^* is represented by a connectivity matrix  where  represents the presence of a regulatory interaction between genes *i* and *j*, and otherwise 0. Consider two GRNs *s^b^* and ; the similarity of the two networks is obtained by the average Hamming distance over all the regulatory connections:(7)

where *d* denotes the Hamming distance and |*s^b^*| denotes the number of connections in the network *s^b^*. In (7), the stability *ρ* ∈ [0, 1], where higher values denote high stability of the GRN inference algorithm. Here, stability is measured with respect to the whole network and the Hamming distance takes into account both the presence and absence of a regulatory connection.

Using the above measures, the stability of complete GRNs is obtained by averaging over *B* number of structures obtained using a particular method. The average of pair-wise stability, such as denoted in (7), is then used as the overall stability performance of the given method. This is given by:(8)

The stability of an independent edge is equally important. With a small perturbation, an edge is either established between two genes *i* and *j*, or is not detected. Hence, based on the number of times a regulatory relationship occurs between the two specific genes, the stability of the edge can be computed. Such edge stability is defined as:(9)

### Implementation

Scale-free network topologies were generated using *igraph* R package [21]. The stability of networks are studied with penalized regression methods. Each of these MVAR method has regularization parameters. Although computationally expensive, we used the leave-one-out cross-validation to estimate the λ for ridge MVAR and *α* and/or λ for sparse models. For ridge estimate, the λ was selected from the set of {0.001, 0.1, 1, 10, 100}. For lasso and elastic net, the glmnet package [22] is used which could generate the whole solution path for λ for a given *α* value (*α* = 1 for lasso). Hence, the *α* values are selected from a set of {0.1, 0.2, …, 0.9} for elastic-net model.

The presence of statistically significant edges were determined using *t-* distribution over regression coefficients corrected for multiple comparisons using false discovery rate (FDR) [23]. In this method, let *P*_(1)_ ≤ *P*_(2)_ ≤ … ≤ *P*_(*n*)_ denotes sorted *p*-values where *n* is the number of hypotheses. If k* is the largest n* for which  then we reject all the hypotheses n* = 1, 2,…, k*. Here, *q* is level of significance. For a given regression, the p-value for a regression coefficient *β* could be approximated using:(10)

Here, *t*(*d*) is *t*-distribution with *d* degree of freedom (dof); σ^2^ is an estimate of the error of variance ; and *w_k_* is the j-th diagonal element of ((*Z′Z* + *λW*^–^)^–1^*Z′Z*(*Z′Z* + *λW*^–^)^–1^) where *W^–^* denotes generalized inverse of *W*, a diagonal matrix with diagonal elements |*β_k_*|. To achieve closed form solution, this matrix is estimated using ridge coefficients using the tuned λ for lasso penalty [24]. For lasso, the number of nonzero coefficients is an unbiased estimate for the degrees of freedom [25]. The dof for elastic-net were also assumed to be the number of nonzero coefficients. Only the statistically significant edges were evaluated for stability analysis of sparse MVAR on real datasets.

In this paper, we use fast coordinate descent algorithm for optimizing lasso and elastic net loss function. The codes are provided with glmnet MATLAB package. Ridge regression is performed using standard MATLAB function.

## Results on synthetic dataset

To study the effects of the number of time points on the stability, GRN consisting of *I* ∈ {10, 50, 100} number of genes were simulated using scale-free networks with topologies having power-law coefficient γ ∈ [ 2.10, 2.30 ]. Thus, temporal gene expressions for *T* ∈ {10, 30, 50, 70} number of time points for a given network topology were generated. For given *I*, γ, and *T*, *B* = 100 number of bootstrapped datasets were generated by randomly initiating gene expressions at the first time point.

Table 2 shows accuracies and stabilities of sparse linear models at different numbers of genes and time points. True positives (TP), false positives (FP), and F-measure (which is equal to , where Precision = TP/(TP+FP) and Recall = TP/(TP + FN) were used to indicate the accuracy. The performances measures reported are the averages over 100 bootstrap datasets. As seen, the accuracies of elastic-net and lasso were good and they need only a number of time points approximately equaling the number of genes to achieve good accuracy in small networks (for example, 10 genes). But as the network size grows, the number of time points required were relatively less compared to the size of the network. Ridge shows relatively poor accuracy, so is not considered further for evaluation. If the number of time points are small, false negatives were easier to correct by increasing the number of time points than false positives.

**Table 2:** Performances of lasso, elastic-net (EN), and ridge MVAR models on synthetic datasets with varying numbers of genes and time points.

Number of genes	Number of time points	Method	True positives	False positives	Precision	Recall	F-measure	Stability
		EN	5.86	7.94	0.47	0.65	0.53	0.41
	**10**	Lasso	**5.19**	**4.88**	**0.57**	0.58	**0.55**	0.46
		Ridge	8.99	90.70	0.09	**1.00**	0.16	**0.99**
	
		EN	8.66	8.02	0.56	0.96	0.69	0.64
	30	Lasso	8.54	5.79	0.63	0.95	0.75	0.65
		Ridge	8.99	90.55	0.09	1.00	0.16	0.99
	
		EN	8.99	5.91	0.64	1.00	0.77	0.71
**10**	50	Lasso	8.99	4.12	0.71	1.00	0.82	0.75
		Ridge	8.98	90.53	0.09	1.00	0.16	0.99
	
		EN	9.00	5.12	0.67	1.00	0.79	0.72
	70	Lasso	8.99	3.92	0.72	1.00	0.83	0.76
		Ridge	8.99	90.35	0.09	1.00	0.16	0.99

		EN	17.29	131.50	0.12	0.35	0.18	0.15
	10	Lasso	13.53	42.81	0.25	0.28	0.26	0.15
		Ridge	48.90	2429.90	0.02	1.00	0.04	0.99
	
		EN	32.73	95.75	0.26	0.67	0.37	0.27
	30	Lasso	32.37	72.41	0.31	0.66	0.42	0.31
		Ridge	48.90	2431.70	0.02	1.00	0.04	0.99
	
		EN	39.39	77.88	0.34	0.80	0.48	0.35
**50**	**50**	Lasso	**39.15**	**0.39**	0.80	**0.82**	**0.52**	0.38
		Ridge	48.9	2436.20	0.02	**1.00**	0.04	**0.99**
	
		EN	48.82	98.11	0.34	1.00	0.50	0.41
	70	Lasso	48.82	78.38	0.39	1.00	0.56	0.45
		Ridge	49.00	2432.90	0.02	1.00	0.04	0.99

		EN	22.33	259.46	0.08	0.23	0.12	0.08
	10	Lasso	14.91	69.95	0.18	0.15	0.16	0.08
		Ridge	98.31	9759.90	0.01	1.00	0.02	0.98
	
		EN	58.67	214.17	0.22	0.59	0.32	0.20
	30	Lasso	56.37	153.76	0.27	0.57	0.37	0.22
		Ridge	98.71	9777.50	0.01	1.00	0.02	0.99
	
		EN	79.10	190.56	0.30	0.80	0.43	0.29
**100**	**50**	Lasso	**78.60**	**164.55**	**0.33**	0.79	**0.46**	0.32
		Ridge	98.80	9792.20	0.01	**1.00**	0.02	**0.99**
	
		EN	87.62	160.01	0.36	0.89	0.51	0.36
	70	Lasso	87.54	144.63	0.38	0.88	0.53	0.38
		Ridge	98.81	9805	0.01	1.00	0.02	0.99

**Figure 1:**
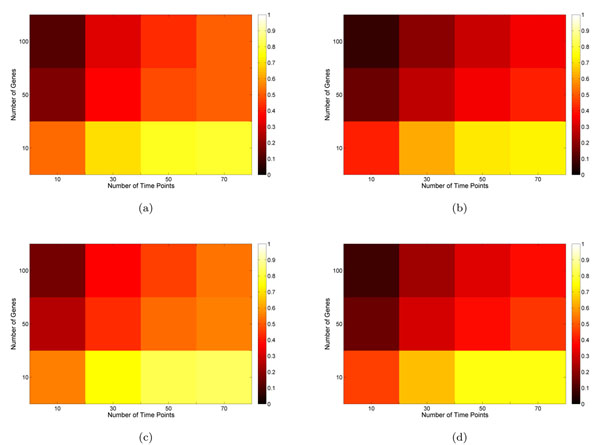
**Stability of networks.** Illustration of the effects of the number of time points on accuracy (F-measure) and stability of sparse MVAR models of building networks of varying number of genes. Figures (a) and (b) denote accuracy and stability of elastic-net penalty respectively while Figure (c) and (d) show accuracy and stability with Lasso penalty respectively.

**Figure 2:**
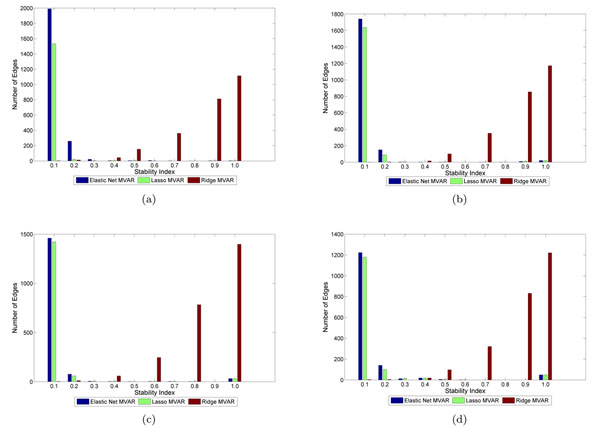
**Distribution of stability of edges.** Distributions of stability of edges with increase in time points on a 50 gene network. Figure (a), (b), (c), and (d) show distribution with 10, 30, 50, and 70 time points respectively.

**Figure 3:**
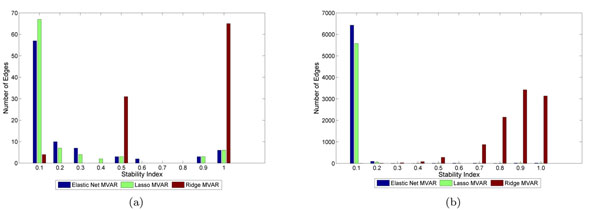
**Distribution of stability of edges**. Distribution of stability of edges with increase in number of genes with 30 time points. Figure (a) and (b) show distribution with 10 and 100 genes respectively.

Figure 1 shows the stability of lasso and elastic-net MVAR methods of building GRN of different sizes with different number of time points. The accuracy and stability were similar for the two methods. To achieve decent accuracy, the number of time points needed were at least about the size of the network. But to achieve good stability, much more time points are needed. At the individual edge level, figure 2 and 3 represent distribution of stability of edges with increasing number of genes and time points. Increase in number of time samples reduces the number of edges of low stability.

## Results on real dataset

**Figure 4:**
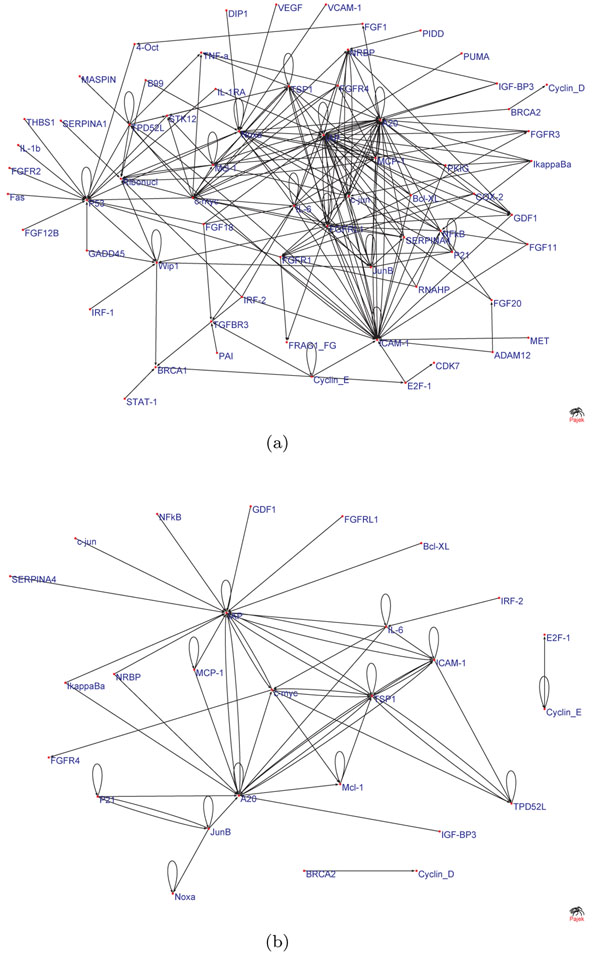
**Gene networks obtained using elastic-net penalty.** GRNs obtained using elastic-net penalty: networks showing (a) all the edges that are statistically significant, and (b) all the edges that have stability greater than 0.5 for SNR = 4.

**Figure 5:**
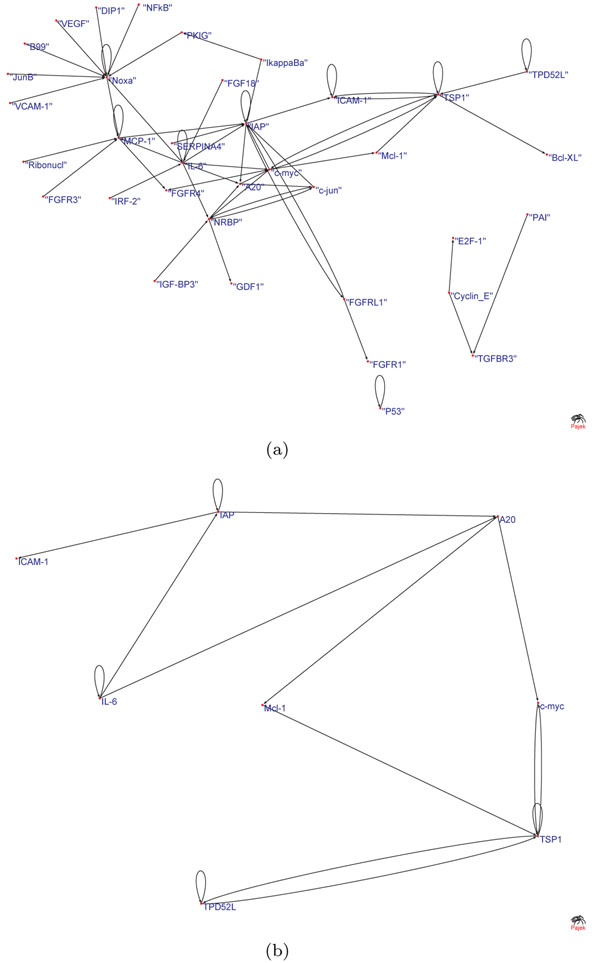
**Gene networks obtained using lasso penalty.** GRNs obtained using lasso penalty:networks showing (a) all the edges that are statistically significant, and (b) all the edges that have stability greater than 0.5 for SNR = 4.

Figures 4a and 5a show statistically significant connections of GRN obtained using elastic-net and lasso MVAR, respectively, for Hela cell-cycle dataset. Figures 4b and 5b show the stable networks whose edges that have stability values greater than 0.5 after perturbations at SNR=4.0. As seen, the network derived using elastic-net penalty has higher number of edges compared to GRNs derived using lasso. This is because lasso can only select a very few number of edges in the presence of correlated gene expressions. As seen, after perturbation, the number of in-degree or out-degree of many critical hubs are reduced while few new edges are also detected. Elastic-net produced a more stable network than the lasso.

**Figure 6:**
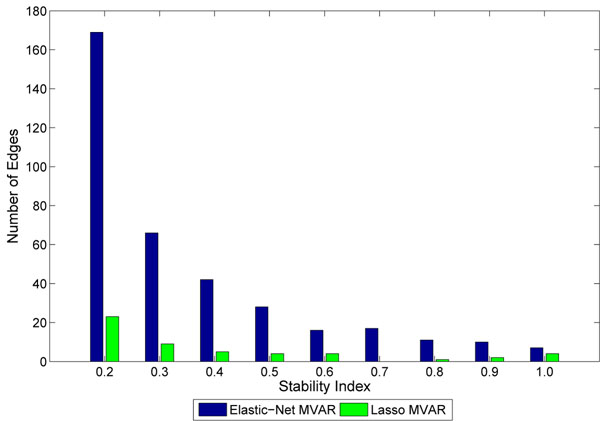
**Distribution of stability of edges on real dataset**. Distribution of stability of edges over perturbed real dataset with SNR = 4 perturbation level

**Figure 7:**
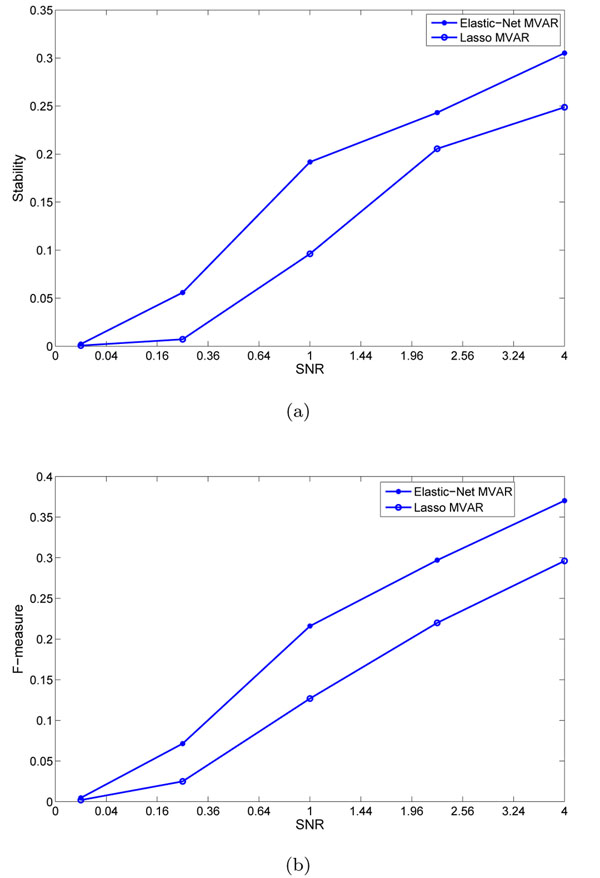
**Stability and F-measure of GRNs derived using puerturbed real dataset**. Figure (a) shows the effects of perturbation on stability while Figure (b) depicts such effect on F-measure of GRNs derived using lasso and elastic-net MVAR on real data collected over Hela cell-cycle.

Both the networks were able to detect many important biological hubs, such as Noxa, IL-6, c-myc, IAP, TSP1 etc. The biological importance of these hubs in cell cycle regulation and tumor developments are well documented in various studies. Briefly, Noxa mediates cell cycle control of homeostasis of B cells and by repressing Noxa, induction of G1 arrest by p18 bypasses a homeostatic cell-cycle checkpoint in intermediate Plasma cells for their differentiation [26]. IL-6 plays importance role in induction of apoptosis and cell cycle regulation [27]. This is also shown by large number of out-degree edges. Thrombospondin-1 (TSP1) curtails tumor growth and acts as an inhibitor of angiogenesis [28]. Inhibitors of apoptosis (IAPs) have important role in cell division and regulates apoptosis [29]. c-Myc oncoprotein prevents cell cycle arrest in response to growth-inhibitory signals, differentiation stimuli, or mitogen withdrawal [30]. Hubs such as P53, NFkB, FGFR3, etc. were not detected in GRN obtained using Lasso penalty, but were detected with elastic-net penalty. However, many edges and hubs are not recognized when perturbations were induced (for example, at SNR = 4.0) and edge stability is set to a thresold of 0.5. Genes like Noxa, NFkB, P53 etc. are severely affected in GRNs obtained using both sparse MVAR methods. Biologically, this could mean that these genes are less important for the biological processes underlying the network, compared to more stable genes. The results indicate that random perturbation of data indirectly helps the process of building accurate and stable GRNs. Figure 6 shows stability of individual connections produced by lasso and elastic-net methods. As seen, lasso produces less number of unstable edges than elastic-net. Figure 7 shows the effect of SNR on stability and F-measure. With increase in SNR level, both the stability and F-measures improves. Generally, the GRNs obtained using elastic-net penalty have higher stability and F-measure compared to GRNs derived using lasso. To compute the F-measure, it is assumed that the GRN derived on real data using lasso (or elastic-net) MVAR is true.

## Discussion

Novel measures of evaluating stability of building GRN were introduced. Thereby, stability and accuracy of sparse MVAR models in building GRN structure were studied using synthetic datasets. The results suggest that to achieve decent accuracy and stability with sparse MVAR methods, at least a number of time points equal to the number of genes are required. But as the network size grows, the number of time points required is less compared to the number of genes in the network. It is easier to ameliorate the effects of false negatives than the false positives by increasing the number of time samples of gene expressions. The results indicates that lasso MVAR and elastic-net perform equally on datasets in general, though lasso handles false positives better. However, elastic-net performed better than lasso on real dataset. This is because elastic-net penalty has an ability to predict regulatory relationship between highly correlated genes while lasso will only predict one of them [31]. As genes are highly correlated, elastic-net or their improved versions proves better making inferences on large scale GRNs [7]. In simulated datasets, correlations among gene expression were not simulated.

To the best of our knowledge, this is the first study introducing stability criteria or studying methods building GRN. Our investigations here were focused on the stability of sparse MVAR models. Our work could be extended for other approaches as well. For small networks, sparse linear models to build networks were stable and accurate. Furthermore, effects of the number of time points on the stability were studied by experimenting on scale-free networks of different topologies. As the network size grows, the number of time points required for building GRN is less than the number of genes in the GRN. Stability is inherent problem in practice as real datasets consist of a large number of genes and short time-series. With an application of real dataset, we demonstrate how stable GRN can be derived by introducing perturbations to the gene expression datasets. Only a few statistically significant edges and associated gene hubs are stable and can withstand small amount of perturbation. Biologically, more stable genes have preserve more significant roles of the biological process of the network. This research emphasizes the need for building GRN that are accurate, stable, and reproducible, so that the structures derived are robust against noise and perturbation of data. In addition by perturbing gene expressions, more accurate and stable core genes and subnetworks can be inferred from temporal gene expression data.

## Competing interests

The authors declare that they have no competing interests.

## Authors' contributions

JR initiated the study, analyzed the results, and wrote the manuscript. PM helped in formulating the experiments, performed all the experiments, and wrote the initial draft. All authors read and approved the final manuscript.
